# Ellagic acid through attenuation of neuro-inflammatory response exerted antidepressant-like effects in socially isolated mice

**DOI:** 10.1016/j.heliyon.2023.e15550

**Published:** 2023-04-17

**Authors:** Zahra Mazrooei, Hossein Tahmasebi Dehkordi, Maryam Hashemi Shahraki, Zahra Lorigooini, Elham Zarean, Hossein Amini-khoei

**Affiliations:** Medical Plants Research Center, Basic Health Sciences Institute, Shahrekord University of Medical Sciences, Shahrekord, Iran

**Keywords:** Ellagic acid, Social isolation stress, Neuroinflammation, Depression

## Abstract

Recent studies have been demonstrated that neuroinflammation plays a crucial role in the pathophysiology of depression. Therefore, anti-inflammatory medications could be regarded as a potentially effective treatments for depression. Ellagic acid (EA) is a natural polyphenol with antioxidant and anti-inflammatory properties. This study aimed to evaluate the antidepressant-like effect of EA in a mouse model of social isolation stress (SIS), considering its potential *anti*-neuroinflammatory properties. In this study, 48 male mice were divided into six groups (n = 8), including saline-treated control (socially conditioned (SC)) group and SIS (isolation conditioned (IC)) groups treated with saline or EA at doses of 12.5, 25, 50, and 100 mg/kg, respectively. Saline and EA were administrated intraperitoneally for 14 constant days. Immobility time in the forced swimming test (FST) and grooming activity time in the splash test were measured. The gene expression of inflammatory cytokines relevant to neuroinflammation was assessed in the hippocampus by real-time PCR. Results showed that SIS significantly increased immobility time in the FST and reduced grooming activity time in the splash test. In addition, the expression of inflammatory genes, including TNF-α, IL-1β, and TLR4 increased in IC mice's hippocampi. Findings showed that EA decreased immobility time in the FST and increased grooming activity time in the splash test. Moreover, EA attenuated neuroimmune-response in the hippocampus. In conclusion, finding determined that EA, through attenuation of neuroinflammation in the hippocampus, partially at least, exerted an antidepressant-like effect in the mouse model of SIS.

## Significance statement

Ellagic acid through attenuation of neuroimmune response in the hippocampus, partially at least exerted an antidepressant-like effect in the mouse model of social isolation stress.

## 1 Introduction

Depression is one of the most common mental disorders in the world. It is estimated that more than 300 million individuals suffer from depression globally [1,2]. Depression is a public health concern significantly linked to functional disability among adults [3]. Besides, it contributes significantly to the global burden of disease [4]. Drug-resistant depression is highly prevalent and closely related to an increased risk of suicide and a worse life quality [5]. Unfortunately, despite the availability of various antidepressants and evidence-based therapies to treat depression, roughly 30% of individuals do not respond sufficiently to therapies [6]. Furthermore, antidepressants have been demonstrated to have adverse side effects [7]. Thus, it has become a challenge for researchers to develop proper pharmacological treatments with the maximum efficacy and lowest adverse effects [8].

The social isolation stress (SIS) model in rodents is a form of chronic psychological stress closely related to induce of depressive-like behaviors [9]. It has been shown that SIS leads to the activation of the hypothalamic-pituitary-adrenal (HPA) and sympathetic-adrenal-medullary (SAM) axis. SIS is associated with an increase in the release of glucocorticoids (GCs), catecholamines, oxytocin, and vasopressin. In several regions of the central nervous system (CNS), SIS alters the level of neurotransmitters such as dopamine, serotonin, gamma-aminobutyric acid (GABA), glutamate, nitric oxide, and adrenaline as well as leads to alteration in receptor sensitivity of *N*-methyl-d-aspartate (NMDA) and opioid system [[10], [11], [12], [13], [14]]. Previous research has found that SIS has long-term and profound effects on rodent's behavior. However, just a few studies have looked into the behavioral effects of SIS in adult mice [15].

Emerging studies suggest that inflammation is involved in developing depression, and there is an association between neuroinflammation and depression [16]. Furthermore, it has been shown that the serum levels of inflammatory cytokines such as interleukin- 1beta (IL-1β), IL-6, and tumor necrosis factor-alpha (TNF-α) are higher in individuals suffering from depression in compared to healthy people [17,18]. Besides, in animal models, researches have shown that inflammatory cytokines increased in the hippocampus of socially isolated rats [19]. Inflammation also contributes to depression symptom, especially neuro-vegetative symptoms, and elevated inflammatory markers are related to poorer treatment outcomes [20]. Surprisingly, increased levels of inflammatory markers in the blood of MDD patients are linked to suicidal thoughts [21]. As a result, anti-inflammatory medications could be regarded a potentially effective treatment for depression [22].

Recently, polyphenolic compounds, which feature one or more hydroxyl groups in their structure, have attracted much consideration due to their effective anti-oxidant properties [23]. Among natural polyphenols, ellagic acid (EA), a component of ellagitannins, is identified for its possible health advantages, such as anti-oxidant, anti-diabetic, neuroprotective, anti-inflammatory, hepato- and cardioprotective, chemo-preventive, anti-cancer, and apoptosis-inducing activities [24,25]. EA is originate abundantly in a series of fruits, vegetables, and nuts either unaccompanied or in the form of ellagitannins, hydrolyzable tannins, and can be extracted by hydrolysis [25]. Accordingly, the acidic hydrolysis of ellagitannins produces EA, a dimeric derived of gallic acid. It's a planar molecule with four hydroxyl groups and two lactone groups [26]. Meanwhile EA has antioxidant possessions because of hydroxyl groups in its structure, it can exert anti-inflammatory activities and play an important role in regulating inflammation and neuroprotection [27]. Therefore, it could be a potential therapy for inflammation-based diseases such as depression.

Accordingly, for the first time, this study was planned to determine the antidepressant-like effect of EA, focusing on its possible role in modulating neuroinflammation in the hippocampus in socially isolated mice.

## 2 Methods

### 2.1 Ethics

This study was implemented in accordance with Guide for the Care and Use of Laboratory Animals (8th edition, National Academies Press) of National Institutes of Health (NIH). All experimental trials in this study were permitted by the Ethics Committee of Shahrekord University of Medical Sciences (Ethical code: IR. SKUMS.REC.1398.024). Every effort was made to diminish animal utilization and heighten animal welfare.

#### 2.1.1 Animals and SIS paradigm

In the current study, male NMRI (Naval Medical Research Institute) mice (Pasteur Institute, Tehran, Iran), weighing 10–12 g and on the postnatal day (PND) 21–23 were used. Each trial group consisted of 8 animals. The animals were housed under standard conditions (temperature: 22 ± 2 °C, 12-h light-dark cycle, and easy access to food and water) for four weeks.

Animals were housed under two different settings, social condition (SC) (control group), and socially isolated (SI). Control mice were housed in groups (six mice per cage) in Plexiglas boxes (25 × 25 × 15 cm), while SI mice were housed individually in Plexiglas boxes (24 × 17 × 12 cm) for 3 weeks. SI mice were housed in a distinct area and had olfactory and visual contact. The cages of SI mice were cleaned weekly by a similar experimenter to circumvent minimum handling and social contact. All trials were carried out between 9:00 a.m. and 01:00 p.m [28].

### 2.2 Experiment design and treatments

Forty-eight male NMRI mice were alienated into 6 groups (n = 8). Group 1 was the control mice that received normal saline (1 ml/kg). Groups 2–6 were the SI mice which received normal saline (1 ml/kg) or EA at doses of 12.5, 25, 50, and 100 mg/kg, correspondingly. All agents were administrated via the intraperitoneal (i.p) route for 14 continuous days. Based on past research and our pilot study dose and time of agent administrations were selected [29,30].

#### 2.2.1 Open-field test (OFT)

In this trial, OFT was used to assess the locomotor behavior of the mice. The OFT device is made of matte white plexiglass (50 cm × 50 cm × 30 cm). Each mouse was put judiciously in the middle square (30 cm × 30 cm), the camera documented its behavior for 5 min, and the data were then investigated using EthoVision software version 8. The horizontal activity (distance traveled) in the OFT was measured [11].

#### 2.2.2 Forced swimming test (FST)

The FST was applied to consider the depressive-like behavior of the mice in addition to screen for new antidepressants. Mice were putted in a cylinder (80 cm in diameter and 25 cm in height) filled with water (1.24 cm and 19 cm). Each mouse was surveyed for motionlessness when it quit struggling and floated motionless on the water, making only movements that kept its head above water. Behaviors were detected in 6-min periods, and the time of immovability was recorded in the last 4 min [11].

#### 2.2.3 Splash test

The splash test was carried out to study the self-motivation and self-care behaviors that specify anhedonia, a depressive-like behavior, in the mice. So as to do this, a 10% sucrose solution was sprayed into the cage on the back of the mouse. The whole grooming actions, including cleaning the nose, face, and head, were chronicled within 5 min after spraying the sucrose solution [11].

#### 2.2.4 Inflammatory gene expression in the hippocampus

Instantaneously afterward behavioral tests, mice were euthanized under profound anesthesia using combination of xylazine 8 mg/kg and ketamine hydrochloride 50 mg/kg, and the hippocampi were remote. The gene expression of TNF-α, IL-1β and TLR4 was estimated by real-time PCR. Toward do this initially, total RNA was isolated from the hippocampus by means of the TRIzol reagent (Invitrogen). qRT-PCR was used to calculate deviations in gene mRNA levels after reverse transcription of 1 μg of RNA from each sample with the PrimeScript RT reagent kit (Takara Bio, Inc., Otsu, Japan). SYBR Premix Ex Taq technique was used for qRT-PCR using a light cycler tool (Roche Diagnostics, Mannheim, Germany) (Takara Bio). The B2m gene was used as a normalizer, and the proportion of alteration in expression of the beleaguered genes was compared to that of the normalizer based on the 2^−ΔΔCt^ qualified expression formula [31]. The genes and their primers are listed in Table 1.

**Table 1:** Primer sequences used in PCR amplification.

Primer	Forward sequence	Reverse sequence
B2m	GGAAGTTGGGCTTCCCATTCT	CGTGATCTTTCTGGTGCTTGTC
TLR4	ATGGCATGGCTTACACCACC	ATGGCATGGCTTACACCACC
TNF-α	TAGCCATTGTGAAGGAGGGC	CCTGAGGCCGTTCCTTGTAG
IL-1β	GCTCCAGCACTATGTCACCA	CGTCTGAGCTGGAAACCAGT

#### 2.2.5 Statistical analysis

The Prism software version 8 was used to consider the collected data. Kolmogorov–Smirnov test was used to measure the normal distribution of data. Normally distributed data are expressed as mean ± S.E.M and were analyzed with one-way analysis of variance (ANOVA) followed by Tukey's post hoc test. P ≤ 0.05 was considered significant.

## 3 Results

### 3.1 Effects of EA on locomotor activity in the OFT

**Fig. 1:**
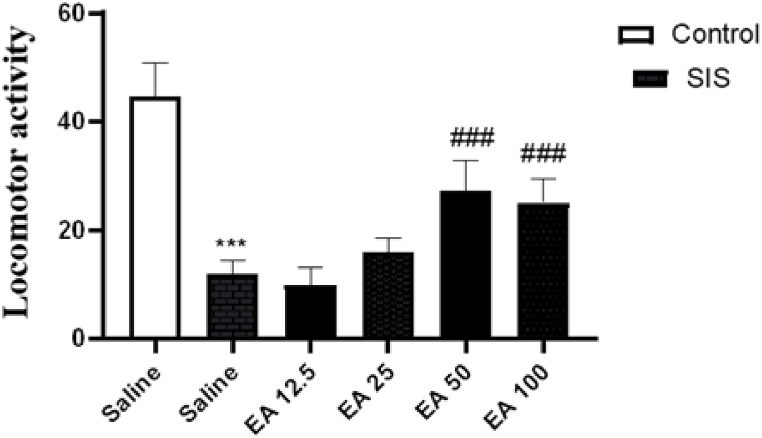
Effect of EA on distance moved in OFT. Values are presented as the mean ± S.E.M from 8 animals and were analyzed using one-way ANOVA followed by Tukey's post-test. ***p < 0.001 in compared with the saline-treated control mice and ###p < 0.001 compared with the saline-treated SIS mice. SIS: social isolation stress, EA: ellagic acid.

Results revealed that SIS significantly lowers the locomotor activity in the experimental group likened to the control group (P < 0.001). Also, treatment with EA at doses of 50 and 100 mg/kg caused a significant rise in locomotor activity in the SIS groups (P < 0.001) in compared to the saline-treated IC group. Though, administration of EA at 12.5 and 25 mg/kg doses showed no significant effect on locomotor activity in SIS groups (Fig. 1).

### 3.2 Effects of EA on immobility time in FST

**Fig. 2:**
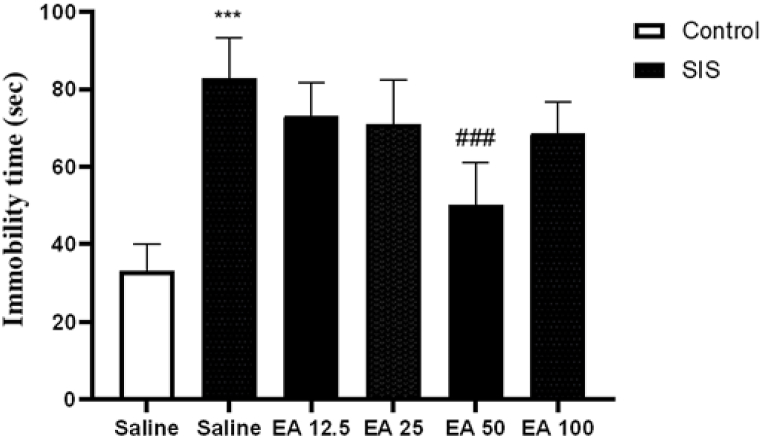
Effect of EA on the period of immobility in the FST. Values are presented as the mean ± S.E.M from 8 animals and were analyzed using one-way ANOVA followed by Tukey's post-test. ***p < 0.001 in compared with the saline-treated control mice and ###p < 0.001 in compared with the saline-treated SIS mice. SIS: social isolation stress, EA: ellagic acid.

Rendering to the results (Fig. 2), SIS caused a significant rise in the immobility time in the experimental group compared to the control group (P < 0.001). Also, the administration of EA at dose of 50 mg/kg to SIS mice meaningfully summary the motionlessness duration in compared to the saline-treated IC group (P < 0.001). Nevertheless, treatment with EA at 12.5, 25, and 100 mg/kg had no significant effects on immobility time in SIS groups in compared to the saline-treated IC mice.

#### 3.2.1 Effects of EA on cleansing time in the splash test

**Fig. 3:**
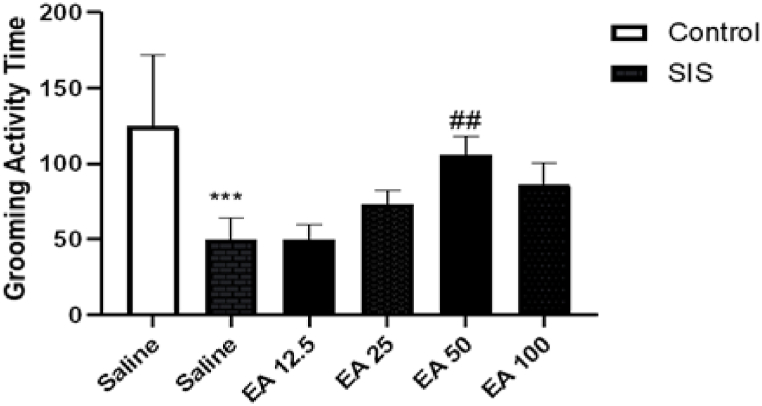
Effect of EA on grooming activity time in splash test. Values are presented as the mean ± S.E.M from 8 animals and were analyzed using one-way ANOVA followed by Tukey's post-test. ***p < 0.001 in compared with the saline-treated control mice and ##p < 0.01 in compared with the saline-treated SIS mice. SIS: social isolation stress, EA: ellagic acid.

Based on the results (Fig. 3), SIS meaningfully reduced the grooming activity period in compared to the control group (P < 0.001). Moreover, treated of SIS mice with EA at the dose of 50 mg/kg suggestively increased the grooming activity time in compared to the saline-treated IC mice (P < 0.01). Treatment with EA at doses of 12.5, 25, and 100 mg/kg had no substantial effects on the grooming activity time in compared to the saline-treated IC mice.

#### 3.2.2 The effect of EA on IL-1β gene expression

**Fig. 4:**
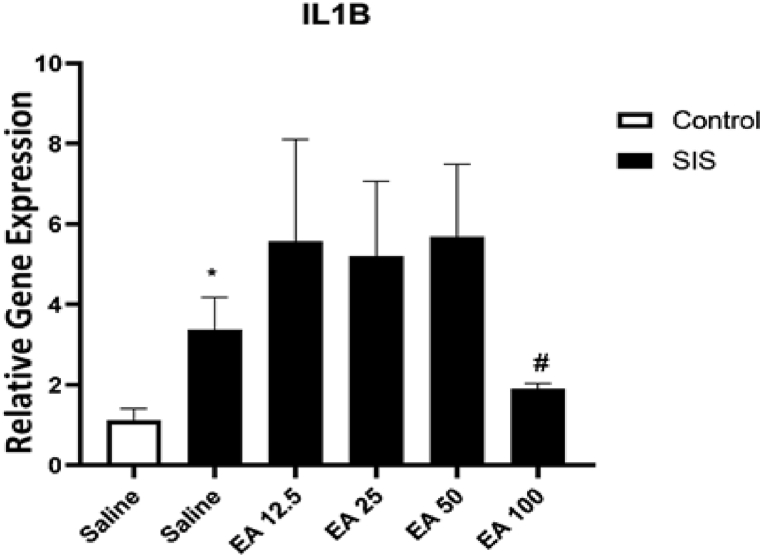
The effect of EA on the gene expression of IL-1β in the hippocampus. Values are presented as the mean ± S.E.M and were analyzed using one-way ANOVA followed by Tukey's post-test. *p < 0.05 in compared with the saline-treated control mice and #p < 0.05 in compared with the saline-treated SIS mice. SIS: social isolation stress, EA: ellagic acid.

According to the findings in Fig. 4, SIS significantly increased the IL-1β gene expression in the hippocampus in compared to the control group (P < 0.05). Besides, treatment of SIS mice with EA at a dose of 100 mg/kg meaningfully condensed the gene expression of IL-1β in the hippocampus in compared to the saline-treated IC mice (P < 0.05). The administration of EA at doses of 12.5, 25, and 50 mg/kg had no important effects on the gene expression IL-1β in the SIS groups in compared to the saline0-traeated IC mice.

#### 3.2.3 The effect of EA on the expression of the TLR4 gene

**Fig. 5:**
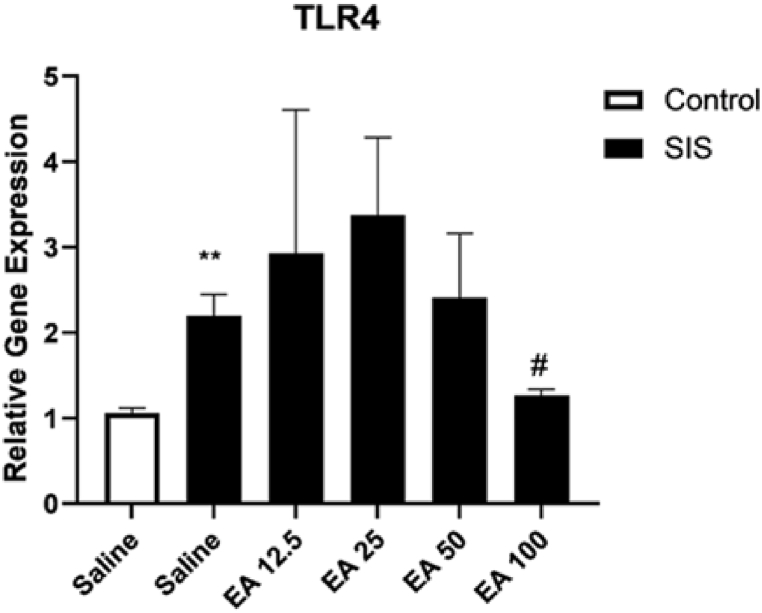
The effect of EA on the gene expression of TLR4 in the hippocampus. Values are presented as the mean ± S.E.M and were analyzed using one-way ANOVA followed by Tukey's post-test. **p < 0.01 in compared with the saline-treated control mice and #p < 0.05 in compared with the saline-treated SIS mice. SIS: social isolation stress, EA: ellagic acid.

The results of the present study showed that SIS produced a significant rise in the gene expression of TLR4 in the hippocampus in compared to the control group (P < 0.01). Treatment with EA at a dose of 100 mg/kg significantly reduced the gene expression of TLR4 in the hippocampus of the SIS mice in compared to the saline-treated IC mice (P < 0.05). The administration of EA at doses of 12.5, 25, and 50 mg/kg had no significant effects on the gene expression of TLR4 in the hippocampus of SIS groups in compared to the saline-treated IC mice (Fig. 5).

#### 3.2.4 The effect of EA on the expression of the TNF-α gene

**Fig. 6:**
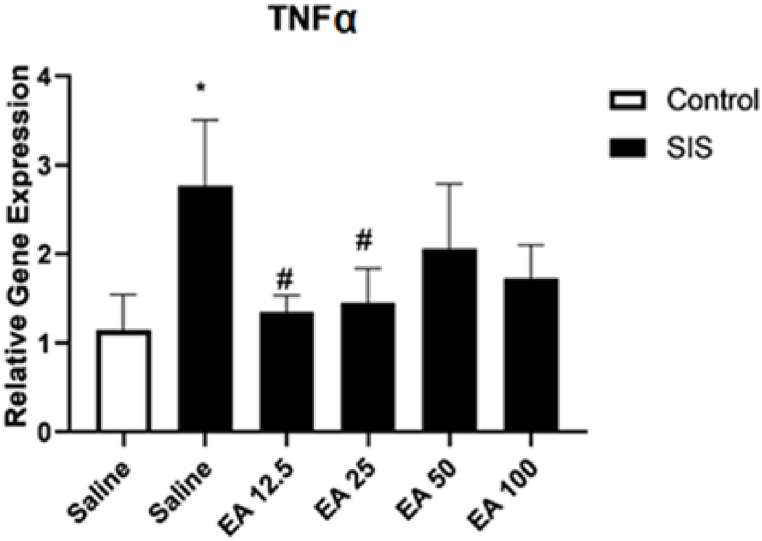
The effect of EA on the gene expression of TNF-α in the hippocampus. Values are presented as the mean ± S.E.M and were analyzed using one-way ANOVA followed by Tukey's post-test. *p < 0.05 in compared with the saline-treated control mice and #p < 0.05 in compared to the saline-treated SIS mice. SIS: social isolation stress, EA: ellagic acid.

Results revealed that SIS produced a substantial increase in the gene expression of TNF-α in the hippocampus in compared to the control group (P < 0.05) (Fig. 6). Also, treatment of SIS mice with EA at doses of 12.5 and 25 mg/kg considerably reduced the gene expression of TNF-α in the hippocampus in compared to the saline-treated IC mice (P < 0.05). The administration of EA at doses of 50 and 100 mg/kg to SIS mice had no significant effects on the gene expression of SIS groups in compared to the saline0treated IC mice.

## 4 Discussion

Outcome demonstrated that SIS irritates depressive-like symptoms such as increased immobility time in the FST and diminished grooming activity time in the splash test. These behavioral alterations were associated with elevated pro-inflammatory gene expression, including TNF-α, TLR4, and IL-1β in the hippocampus. Treatment with EA attenuated the negative effects of SIS on behaviors as well as mitigated neuro-immune response in the hippocampus.

Evidences showed that SIS could induce depressive-like behavior in some behavioral tasks [11]. Based on previous studies, FST and splash test were widely used to assess depression-like behaviors [32]. Ample evidence have demonstrated that SIS is associated with increase in immobility time in the FST and decrease in grooming activity time in the splash test [33,34]. In this experiment, socially isolated have higher immobility time in the FST and lower grooming activity time in the splash test in compared to the control mice.

Evidence suggests that elevated pro-inflammatory cytokines is linked to the depressive symptoms [35]. Preceding studies have determined that oxidative stress and neuro-inflammatory response in the brain is associated with behavioral disorders such as depression [[36], [37], [38], [39]]. Additionally, anti-inflammatory therapies has been demonstrated to reduce depression-like behaviors [40,41], signifying a causal link between neuro-immune response and depression. Previous studies have determined that SIS is associated with neuro-immune response and neuroinflammation in the brain [42,43]. In line with abovementioned studies, finding of the present study showed the gene expression of inflammatory cytokines which are related to neuroinflammation including TNF-α, TLR4 and IL-1β significantly increased in the hippocampus of socially isolated mice. These finding determined that, partially at least, SIS via triggering of neuro-immune response in the hippocampus induce depressive-like behaviors.

EA is a polyphenol compound that displays an extensive range of pharmacological activities counting anti-inflammatory effects [44]. In addition, it has been shown that EA reduced mitochondrial dysfunction and possesses potent neuroprotective possessions [45]. Earlier studies have established that EA possessed antidepressant-like properties [25,29,46]. Indications showed that EA may through modulating of brain derived neurotrophic factor (BDNF), *N*-methyl d-aspartate (NMDA) and nitric oxide (NO) exerted antidepressant-like effects [29,47]. Though full and exact underlying mechanisms are still unknown. Therefore, this experiment carried out to investigate the antidepressant-like effects of EA on the mouse model of SIS regarding its anti-inflammatory properties. Numerous studies have stated anti-inflammatory possessions for EA in different models of inflammatory states [48,49]. Finding of the current study displayed that administration of EA upturned the negative effects of SIS on behavior. Results showed that EA reduced the motionlessness time in the FST and augmented the grooming activity time in the splash test. These finding determined that EA exerted antidepressant-like effect in IC mice. Previous studies have showed that attenuation of neuroinflammatory response in the hippocampus is involved in the antidepressant-like effect of some agents [37,50,51]. Result of the present study determined that treatment of IC mice with EA lessened the neuro-immune response in the hippocampus. EA reduced the gene expression of TNF-α, TLR4 and IL-1β which are related to neuroinflammation in the hippocampus of IC mice. These finding demonstrated that, partially at least, EA via mitigation of neuro-immune response in the hippocampus exerted antidepressant-like effect in socially isolated mice.

## 5 Conclusions

In conclusion, this study verified that EA partly at least, through diminution of neuroinflammatory response in the hippocampus alleviated the depressive-like behaviors following SIS in male mice.

## Author contribution statement

Zahra Mazrooei; Hossein Tahmasebi Dehkordi: Performed the experiments; Wrote the paper.

Maryam Hashemi: Analyzed and interpreted the data.

Zahra Lorigooini; Elham Zarean: Performed the experiments.

Hossein Amini: Conceived and designed the experiments; Contributed reagents, materials, analysis tools or data; Wrote the paper.

## Data availability statement

Data included in article/supplementary material/referenced in article.

## Declaration of interest's statement

The authors have no conflicts of interest to declare regarding the study described in this article and preparation of the article.

## Additional information

No additional information is available for this paper.

## Limitation of study

Limitations of this study include the small number of mice, the use of only male mice, use EA at limited doses, and that behavioral response and gene expression were assessed 2 weeks after treatment.

## Declaration of competing interest

The authors declare that they have no conflicts of interest.
