# Corrigendum to “Ellagic acid through attenuation of neuro-inflammatory response exerted antidepressant-like effects in socially isolated mice” [Heliyon Volume 9, Issue 4, April 2023, Article e15550]

**DOI:** 10.1016/j.heliyon.2025.e43342

**Published:** 2025-04-16

**Authors:** Zahra Mazrooei, Hossein Tahmasebi Dehkordi, Maryam Hashemi Shahraki, Zahra Lorigooini, Elham Zarean, Hossein Amini-khoei

**Affiliations:** Medical Plants Research Center, Basic Health Sciences Institute, Shahrekord University of Medical Sciences, Shahrekord, Iran

In this article, 3 citations [32,44,47] were duplicated references in the article. Reference 34 is a duplication of reference 28. Reference 42 is a duplication of reference 13. Reference 47 is a duplication of reference 46.

The original references can be found below:

*[13] S. Amiri, A. Haj-Mirzaian, H. Amini-khoei, A. Shirzadian, M. Rahimi-Balaei, A. Razmi et al., Lithium attenuates the proconvulsant effect of adolescent social isolation stress via involvement of the nitrergic system, Epilepsy Behav. 61 (2016) 6–13*.

*[18] Y. Jia, L. Liu, C. Sheng, Z. Cheng, L. Cui, M. Li et al., Increased serum levels of cortisol and inflammatory cytokines in people with depression, J. Nerv. Ment. Dis.207 (4) (2019) 271–276*.

*[34] S. Amiri, H. Amini-Khoei, A. Haj-Mirzaian, M. Rahimi-Balaei, P. Naserzadeh, A. Dehpour et al., Tropisetron attenuated the anxiogenic effects of social isolation by modulating nitrergic system and mitochondrial function, Biochim. Biophys. Acta Gen. Subj. 1850 (12) (2015) 2464–2475*.

*[42] S. Amiri, A. Haj-Mirzaian, H. Amini-Khoei, A. Shirzadian, M. Rahimi-Balaei, A. Razmi et al., Lithium attenuates the proconvulsant effect of adolescent social isolation stress via involvement of the nitrergic system, Epilepsy Behav. 61 (2016) 6–13*.

*[46] H.A. Bedel, C. Kencebay Manas, G. Ozbey, C. Usta, The antidepressant-like activity of ellagic acid and its effect on hippocampal brain derived neurotrophic factor levels in mouse depression models, Nat. Prod. Res. 32 (24) (2018) 2932–2935*.

*[47] H.A. Bedel, C. Kencebay Manas, G. Özbey, C. Usta, The antidepressant-like activity of ellagic acid and its effect on hippocampal brain derived neurotrophic factor levels in mouse depression models, Nat. Prod. Res. 32 (24) (2018) 2932–2935*.

The reference list has been ammended to remove the duplicated references.

Reference 34 has been removed from the list as it is a duplicate of reference 28 (S. Amiri, H. Amini-Khoei, A. Haj-Mirzaian, M. Rahimi-Balaei, P. Naserzadeh, A. Dehpour et al., Tropisetron attenuated the anxiogenic effects of social isolation by modulating nitrergic system and mitochondrial function, Biochim. Biophys. Acta Gen. Subj. 1850 (12) (2015) 2464–2475.) All citations to reference 34 should now be associated with reference 28.

Reference 42 has been removed from the list as it is a duplicate of reference 13 (Amiri S, Haj-Mirzaian A, Amini-Khoei H, Shirzadian A, Rahimi-Balaei M, Razmi A et al. Lithium attenuates the proconvulsant effect of adolescent social isolation stress via involvement of the nitrergic system. Epilepsy & Behavior. 2016; 61:6–13.) All citations to reference 42 should now be associated with reference 13.

Reference 47 has been removed from the list as it is a duplicate of reference 46 (Bedel HA, Kencebay Manas C, Özbey G, Usta C. The antidepressant-like activity of ellagic acid and its effect on hippocampal brain derived neurotrophic factor levels in mouse depression models. Natural product research. 2018; 32(24):2932-5.). All citations to references 47 should now be associated with reference 46.

The ***authors*** apologize for the errors.

## Declaration of competing interest

The authors have no conflicts of interest to declare regarding the study described in this article and preparation of the article.

